# Identification of the Aggregation-sex Pheromone of the Cerambycid Beetle *Phymatodes pusillus* ssp. *pusillus* and Evidence of a Synergistic Effect from a Heterospecific Pheromone Component

**DOI:** 10.1007/s10886-018-1008-3

**Published:** 2018-08-28

**Authors:** Mikael A. Molander, Mattias C. Larsson

**Affiliations:** Unit of Chemical Ecology, Department of Plant Protection Biology, Swedish University of Agricultural Sciences, Box 102, Sundsvägen 14, 230 53 Alnarp, Sweden

**Keywords:** Longhorn beetle, 1-hexanol, 2-methyl-1-butanol, Population monitoring, Threatened species, Conservation

## Abstract

The longhorn beetle *Phymatodes* (*Poecilium*) *pusillus* ssp. *pusillus* is a rare, elusive species that is included on Red Lists of threatened species. Previously, 1-hexanol and 1-butanol were reported as putative components of the aggregation-sex pheromone of this species, but behavioral assays to confirm this have not been performed. In this study, we undertook a comprehensive examination of *P. p. pusillus* to verify the presence of a pheromone. Adult beetles were reared from colonized wood and used for headspace sampling. Analyses by gas chromatography-mass spectrometry revealed that two compounds were present in large quantities in the extracts of males, but absent in extracts from females. Male and female antennae showed repeatable responses to the two compounds in electrophysiological recordings. Using synthetic standards, we were able to identify the compounds as 1-hexanol and 2-methyl-1-butanol. A field bioassay demonstrated that the two compounds were unattractive when applied singly, but elicited significant attraction of female and male beetles when applied in blends of different ratios. We also found that the species exhibited significant attraction to a blend of 3-hydroxy-2-hexanone and 2-methyl-1-butanol, which is the aggregation-sex pheromone of at least two closely related and sympatric species. The presence of the heterospecific component 3-hydroxy-2-hexanone synergized a response to 2-methyl-1-butanol. The pheromone of these species may function as a host cue for *P. p. pusillus* as the three species have similar phenology and substrate demands. The aggregation-sex pheromone of *P. p. pusillus* can be used for population monitoring and as a tool to study the general ecology and conservation requirements of this rare species.

## Introduction

Longhorn beetles (Cerambycidae) are a diverse, important group of insects from an ecological and economic point of view (Allison et al. 2004; Evans et al. 2007; Nearns 2013). Thus far, about 35,000 species have been described (Švácha and Lawrence 2014). The larvae are primarily xylophagous, developing under bark and inside woody plant tissues where they function as decomposers and contribute to nutrient cycling (Monné and Monné 2017). Larvae and adult cerambycids are also an important source of food for other wildlife, such as woodpeckers (e.g. Hogstad and Stenberg 1997), and the larval galleries provide micro habitats that are utilized by further species (Buse et al. 2008). Most species colonize trees that are already dead or weakened and have little direct impact on human activities such as forestry. However, a number of longhorn beetles are problematic pest species that occupy healthy, living trees and can severely weaken or kill the infested hosts with great economic consequences (Allison et al. 2004; Faccoli and Gatto 2015; Hanks and Millar 2016).

Knowledge on the chemical ecology of longhorn beetles has seen significant expansion in recent years, particularly in terms of the number of pest, and potential pest, species with identified sex and aggregation-sex pheromones (Hanks and Millar 2016; Millar and Hanks 2017). However, more recently the possibility of exploiting pheromones as a tool to study rare species of conservation concern has been highlighted (Ray et al. 2014; Žunič Kosi et al. 2017). Studies of elusive saproxylic insects that are difficult to sample with conventional survey methods have shown that pheromone-based trapping methods can greatly facilitate ecological studies on these insects (Andersson et al. 2014; Kadej et al. 2015; Musa et al. 2013; Oleksa et al. 2015). Data from field studies that utilize pheromone-based sampling methods to study threatened insect species have been used for key tasks such as population estimates, analysis of species’ landscape composition requirements and mark-recapture studies (Larsson and Svensson 2009, 2011; Musa et al. 2013; Svensson et al. 2011). Inferences from such studies can be used to design and direct conservation efforts. However, pheromones have so far only been identified for a small number of species of conservation concern (Larsson 2016).

*Phymatodes pusillus* ssp. *pusillus* (Fabricius, 1787) is a cerambycid species (subfamily Cerambycinae, tribe Callidiini, formerly in the genus *Poecilium*) of great potential interest to conservation biology. It is considered rare and local throughout its distribution in south and central Europe (Bílý and Mehl 1989; Ehnström and Holmer 2007; Harde 1966; Hellrigl 2010; Jeniš 2001; Klausnitzer et al. 2016; Löbl and Smetana 2010; Niehuis 2001). In Sweden, it is known from a small area in the southern part of the country, which harbors the only known population in northern Europe (Lindhe et al. 2010). The species is considered threatened (threat category vulnerable, VU) on the Swedish national Red List, due to the small area of occupancy and an expected ongoing reduction in the area and quality of its habitat (ArtDatabanken 2015). In addition, *P. p. pusillus* is included in a national action plan issued by the Swedish Environmental Protection Agency for threatened species of longhorn beetles associated with oak (Franc 2013). It is also considered threatened in Germany and Austria (Binot et al. 1998; Jäch 1994), and possibly threatened in Switzerland (category data deficient, DD) (Monnerat et al. 2016). Furthermore, the species is perceived as an indicator of important areas for longhorn beetles in Europe (Jeniš 2001) and of sites rich in other saproxylic insects that are dependent on thin, dead oak branches (Franc 2013).

The chemical ecology of *P. p. pusillus* was studied previously by Schröder (1996) who reported large quantities of 1-hexanol and trace amounts of 1-butanol and 1-octanol in extracts of volatiles from male *P. p. pusillus*. The compounds were not present in the extracts from female beetles and have thus been assumed to be part of a male-produced aggregation-sex pheromone, but behavioral assays to confirm this have not yet been reported. While conducting field studies on other, closely-related cerambycid species we noted an attraction of *P. p. pusillus* to traps with lures consisting of a blend of 3-hydroxy-2-hexanone and 2-methyl-1-butanol (unpublished data), which was surprising as neither of these two compounds had been reported by Schröder (1996).

In the present study, we performed an in-depth investigation of the pheromone of *P. p. pusillus* for the purpose of obtaining a tool for surveying and monitoring populations of this rare and transient species.

## Methods and Materials

### Study Species

*Phymatodes pusillus* is separated into four subspecies (Danilevsky 2018). The present work examined the nominal subspecies, *Phymatodes pusillus* ssp. *pusillus*, which is the only subspecies found in northern and western Europe. The other three subspecies occur in east and southeast Europe (Danilevsky 2018; Löbl and Smetana 2010). Larvae of *P. p. pusillus* develop for one or two years in fresh, recently dead, thin oak branches of a diameter of about two to five centimeters (Ehnström and Holmer 2007; Palm 1953, 1959). The species is xero-thermophilus, inhabiting oak forests in areas with a warm, dry climate (Jeniš 2001; Klausnitzer et al. 2016; Niehuis 2001). Adult beetles can be observed from mid-April to early June while sitting on oak branches that are suitable for oviposition, particularly in sun exposed conditions (Ehnström and Holmer 2007; Lindhe et al. 2010). However, adults of the species are only occasionally observed directly in the field and only about 35 adult individuals have been collected by hand since the species was first discovered in Sweden in 1951 (Lindhe et al. 2010; Palm 1953). The majority of records are of individuals reared from collected wood substrate.

### Study Area

Most observations of *P. p. pusillus* in Sweden are from Ecopark Hornsö (center coordinates: DD 57.0120/16.0897) (Lindhe et al. 2010). Hence, we conducted all of our field work within this 9200 ha area with a mixture of protected semi-natural forests and production stands. Situated in southeastern Sweden, the region has an average yearly temperature of 7.7 °C, receives a total precipitation of 480 mm and about 1700 hr of sunshine per year (SMHI 2017). Ecopark Hornsö is designated as one of the most important areas in northern Europe for saproxylic beetles and harbors populations of numerous threatened species of conservation concern (Lundberg 1993; Nilsson 2001; Nilsson and Huggert 2001). The forests are strikingly diverse with mixed deciduous and coniferous trees, particularly oak, birch, aspen, and Scots pine. Oak is noticeably abundant throughout the park (Nilsson and Huggert 2001). Remarkably, many stands are dominated by older trees and have only been logged to a limited extent in recent decades. Numerous lakes, wetlands and streams add complexity to the landscape. Large areas consist of open, sunlit forests on rocky ground cover resulting in a comparatively dry, warm climate that presumably favors many of the rare insect species, including *Phymatodes p. pusillus*. Modern forestry is practiced in most forest stands, but special biodiversity-friendly measures are used throughout the area, such as retention of old trees and limited harvest of twigs and branches for biofuel production (Anonymous 2008).

### Collection of Insects

To obtain beetles for headspace sampling and electroantennographic studies, we collected oak branches from a stack of biofuel material in the Ecopark (see above), which contained oak branches and thinner logs cut in the winter of 2013 to 2014. The material had been left to dry in sunny conditions at a forest edge over the summer season of 2014, during which many saproxylic insect species had colonized the wood. In February 2015, we collected about 1.5 m^3^ of loosely aggregated branches from the pile. During the following days, the material was transported back to the laboratory, cut into smaller pieces of about 0.5 m length and placed in transparent plastic boxes (dimensions L × W × H: 56 × 39 × 42 cm). Part of each lid was cut open and covered with a fine, plastic mesh to let moisture escape the containers. The boxes were moved into a greenhouse with an average daily temperature of approx 15 °C. Boxes were examined visually twice daily for recently emerged beetles, which tended to reach for the sunlight and climb to the lid or corners of the boxes. The wood material was occasionally dosed with water to reduce desiccation. The first individual of *P. p. pusillus* emerged from the wood after one week. Over the following days an additional seven individuals emerged (in total four females and four males). The beetles were quickly removed from the boxes and placed in small plastic containers (males and females held separately) in a refrigerator at 8 °C. A piece of moist paper was added to each container to prevent dehydration.

### Collection of Volatiles

Beetles were moved from their storage in the refrigerator to a climate chamber the day before an aeration was to start the next morning. This was not the same chamber as the one where the aerations took place, but the settings were identical. In the chamber, males and females were kept separately overnight in two plastic jars with pieces of fresh, dead oak branches and a piece of paper drenched in honey water, which the beetles would occasionally feed on. The following morning, immediately before starting aeration, the beetles were transferred to empty gas washing bottles (1 L; Lenz Laborglas Gmbh, Wertheim, Germany). Besides two bottles containing the males and the females respectively, an empty gas bottle was used as a blank control. The three glass bottles were then moved to a different climate chamber (25 °C constant temperature, light hours 08:30 to 21:00 hr) where the aeration was started.

The adsorption columns consisted of Teflon® (TFE) tubing (inner diameter 3 mm, length 50 mm) containing the adsorbent polymer Porapak™ Q (25 mg; 50–80 mesh; Supelco/Sigma-Aldrich, Munich, Germany) held in place with polypropylene wool, secured by short pieces of smaller Teflon® tubing (inner diameter 1.5 mm, length 2 mm) inserted into the main column on both sides of the adsorbent material. To reduce the presence of other volatile compounds, identical adsorption columns were used during the majority of the aerations to clean the ambient air being pulled into the bottles that contained the beetles. Several sets of adsorption columns were used, which were rotated between different aerations.

Teflon® thread sealing tape was used to attach one adsorption column to each of the three gas washing bottles. Polyvinyl chloride (PVC) tubing (diameter 4 mm) connected the three columns to a single air pump (model PM 10879 NMP 03; KNF Neuberger, Freiburg, Germany) that would pull ambient air through the columns and gas bottles. A triple airflow meter and plastic valves on the PVC tubing were used to adjust the air flow at all three columns simultaneously and achieve a constant flow rate of about 0.25 l/min through each column. The gas bottles were reused, but thoroughly rinsed with ethanol and acetone between aerations and left to dry overnight. Males and females were put in different bottles at different aerations. Aerations lasted for 5 hr between approximately 10.00 and 15.00 hr, the hours when the beetles appear to be the most active in the field under natural conditions. In total, six aeration sessions were performed using this methodology.

The columns were extracted immediately after an aeration using 300 μl of hexane. Occasionally, nitrogen gas was used to gently help push the solvent through the columns. Samples were stored in 1.5 ml screw neck glass vials with closed butyl/PTFE seal screw caps (articles 11,090,210 and 08151653, Skandinaviska Genetec AB, Stockholm, Sweden) at −18 °C until analysis. Finally, the adsorption columns were rinsed by treating them with an additional 3 × 300 μl of hexane followed by 3 × 300 μl of acetone before reuse.

### Pheromone Identification

Analysis of the aeration samples was performed using two systems of coupled gas chromatography-mass spectrometry (GC-MS). The first system (GC model 7890B and MS model 5977A, Agilent Technologies, Palo Alto, CA, USA) was equipped with a DB-WAX column (60 m × 0.25 mm inner diam., d.f. 0.25 μm; J&W Scientific, Folsom, CA, USA). The second system (GC 6890 N and MS 5975, Agilent Technologies) was equipped with an HP-5MS column (60 m × 0.25 inner diam., d.f. 0.25 μm, Agilent Technologies). An aliquot of each aeration sample (2 μl) was injected manually in splitless mode (injector temperature 225 °C on both GCs) with helium as the carrier gas at constant flow rates of 1.9 ml min^-1^ (DB-WAX) and 1.8 ml min^−1^ (HP-5MS) respectively. Front inlet pressures were 182 kPa (DB-Wax) and 172 kPa (HP-5MS). The GC oven temperature programs started at 30 °C, with a 3 min hold, thereafter increasing by 8 °C min^−1^ to 230 °C where the temperature was held for 10 min. However, for the HP-5MS column, we used a maximum end temperature of 260 °C. The mass spectrometers were set to start recording after a 6.5 min solvent delay.

Sex-specific peaks were recognized by visually comparing the chromatograms from males, females and the blank control. Identification of potential pheromone compounds was conducted by matching the mass spectra to commercial database libraries (NIST and Wiley). To verify the identity of the candidate compounds, we compared their GC retention times and mass spectra with those of synthetic standards (1-hexanol, reagent grade 98%, CAS number 111–27-3, Sigma-Aldrich; racemic 2-methyl-1-butanol, **≥** 99% purity, CAS number 137–32-6, Sigma-Aldrich).

### Electrophysiology

Antennal responses of male and female *P. p. pusillus* to volatiles from male beetles were studied using gas chromatography coupled to electroantennographic detection (GC-EAD) with an EAG apparatus (IDAC-2; Syntech, Kirchzarten, Germany) and an Agilent Technologies 7890A GC (DB-WAX column, 30 m × 0.25 mm inner diam., d.f. 0.25 μm; J&W Scientific, Folsom, CA, USA) with flame ionization detection. The GC oven was programmed to start at 30 °C, with a 3 min, and then increasing by 20 °C min^−1^ to 225 °C, where it was held for 10 min. Aliquots of the extracts (2 μl) were injected with hydrogen as the carrier gas at a constant flow rate of 2.1 ml min^−1^. At the GC effluent, 4 psi of nitrogen was added and split 1:1 in a Gerstel 3D/2 low dead volume four-way-cross (Gerstel, Mülheim, Germany) between the flame ionization detector and the EAD. The GC effluent capillary for the EAD passed through a Gerstel ODP-3 transfer line, which tracked the GC oven temperature, into a glass tube (30 cm length, 0.8 cm diam.), where it was continuously mixed with a charcoal-filtered, humidified airstream (18–20 °C, 50 cm s^−1^). Once prepared, the beetle antenna was positioned 0.5 cm from the outlet of the glass tube.

The beetles were kept in the climate chamber for a few hours before starting the electrophysiology studies. A razor blade was used to cut the entire head off from the thorax at the joint behind the occipital foramen. The head was quickly moved to a small droplet of Beadle-Ephrussi Ringer solution to avoid desiccation at the cut joint. Two glass micro capillaries were filled with Ringer solution and mounted on silver wires as the recording electrode and the reference electrode. The recording electrode was connected to a pre-amplifier probe (EAG combi probe, Syntech) connected to a high-impedance DC amplifier interface box (IDAC-2, Syntech). The head of the beetle was mounted on the capillary of the reference electrode and between one to two distal segments of one of the antennae cut off with a pair of micro scissors. Half a segment of the cut antenna was moved into the capillary containing the recording electrode. The setup was then manipulated until a stable connection had been obtained, evidenced by apparent but limited baseline noise typically characterizing a physiologically active preparation. In most cases the preparation remained active and with noise levels within acceptable limits during the whole GC run. A total of six successful recordings were conducted with this method from two male and four female beetles.

### Field Bioassay

Six blends of 1-hexanol and 2-methyl-1-butanol were tested in field trapping tests. Two treatments tested the compounds 1-hexanol and 2-methyl-1-butanol separately at a dose of 50 mg per bait. Four treatments tested the following blends; (1) 6.25 mg 2-methyl-1-butanol and 50 mg 1-hexanol, (2) 12.5 mg 2-methyl-1-butanol and 50 mg 1-hexanol, (3) 25 mg 2-methyl-1-butanol and 50 mg 1-hexanol, (4) 50 mg 2-methyl-1-butanol and 50 mg 1-hexanol. We also added a treatment with a blend of 50 mg racemic 3-hydroxy-2-hexanone (CAS number 54123–75-0, Bedoukian Research, Danbury, CT, USA) and 10 mg 2-methyl-1-butanol, the blend that we had previously found to be attractive to the species in the field (see Introduction). The blends were dissolved in isopropanol (0.5 ml per bait) and transferred to a polyethylene Grippie® zip-lock bag (5.5 × 6.5 cm × 40 μm, Grippie Light Nr-02, b.n.t. Scandinavia AB, Arlöv, Sweden) with a pipette in the field. The bag was carefully sealed and attached to the central part of the trap using metal wire, always on the south-facing side of the trap. Isopropanol (0.5 ml) was used as blank control.

For the bioassay, we located four areas within Ecopark Hornsö in 2016 where the traps would be situated in sunny conditions during a large part of the day. The sites were typically forest edges bordering small, grazed areas or former clear cuts where the new forest had not yet grown tall. At each area, eight traps (one with a lure of each treatment type) were put up and bait added on the 30th of April and 1st of May (two replicates per day). A minimum distance of at least 9 m between two traps was used. The traps were emptied the first time on the 14th and 15th of May (two replicates per day). At the same time, new baits were added and the traps were re-randomised at each site to create a new replicate. All traps were emptied a second time on the 5th of June and the bioassay terminated as this is about the time when the activity period of *P. p. pusillus* is at an end in Sweden (Lindhe et al. 2010).

Traps were custom-made flight-intercept traps with cross-vane panels. The black panels had a length of 25 cm and height of 20 cm (Nordic Plastics Group AB, Trelleborg, Sweden) and the black funnel a diameter of 20 cm and slope of about 45 ° (Hall Miba, Alvesta, Sweden). A brown circular roof (diameter 28 cm) was used as a top cover for rain water protection (Soparco, Chaingy, France). A white trap jar with a volume of one liter (Corning Life Science, Stockholm, Sweden) was attached to the bottom of the funnel and 250 ml of propylene glycol added as a preservative. Cable ties and metal wire was used to hold the different parts together. The panels and the inside of the funnel were coated with a layer of Fluon® (polytetrafluoroethylene dispersion, 60 wt% in H_2_O, Sigma-Aldrich, St. Louis, Missouri, USA), further diluted 1:1 with water, to increase trap efficiency (Graham and Poland 2012). The traps were attached to bars of reinforcement steel with length 2 m and diameter 0.8 cm. The top 25 cm was bent at a 90 ° angle creating a branch from which the trap could hang once tied with metal wire. The bars were forced into the ground to a depth of about 10 cm, so that the center of the trap (and the location of the lure) was situated at about 1.5 m above ground.

Emptying the traps was performed by pouring the propylene glycol with the trapped insects into a tea filter. The fluid was transferred back to the trap jar for reuse, while the filter with the insects was saved in a small plastic bag. The samples were brought back to the laboratory and the numbers of male and female *P. p. pusillus* per sample were counted, based on the color of the abdomen, which is orange-reddish in females and black in males (Ehnström and Holmer 2007). A few individuals were difficult to sex reliably using this method, probably due to discoloration by the propylene glycol, and these were dissected and their genitalia studied to confirm the sex. Finally, the beetles were preserved in 50 ml Falcon™ tubes filled with 70% ethanol for long-term storage. Voucher specimens will be deposited in the Lund entomological collections (Biological Museum, Lund University, Sweden) after further molecular studies.

Furthermore, in 2016 and 2017 we undertook large-scale landscape surveys of *P. p. pusillus* and other species of longhorn beetles at a number of sites in southeastern Sweden (unpublished data). At each site, we used three traps with lures of 50 mg racemic 3-hydroxy-2-hexanone and 10 mg racemic 2-methyl-1-butanol and three traps with lures of 50 mg 1-hexanol and 25 mg racemic 2-methyl-1-butanol. All traps were active throughout the species activity period from late April to early June, and emptied once in mid-May when new lures were added. *P. p. pusillus* was shown to occur at 16 sites in 2016, eight of which were situated within Ecopark Hornsö. We used the *P. p. pusillus* from this landscape study to compare the average number of individuals of each sex per trap that were captured with the two different blends.

In 2017, we continued our landscape study from 2016 with the same methodology and took the opportunity to determine the relative attractiveness of 3-hydroxy-2-hexanone as a single component, by setting one trap with a lure of 50 mg racemic 3-hydroxy-2-hexanone at twelve different locations within Ecopark Hornsö. The catch of *P. p. pusillus* in the traps with 3-hydroxy-2-hexanone as a single component was compared to that of the eight landscape survey sites within the Ecopark, which had a total of 24 traps with lures of 50 mg racemic 3-hydroxy-2-hexanone and 10 mg racemic 2-methyl-1-butanol. All traps in the landscape study of 2017 were deployed between the 27th and 29th of April and emptied a first time (with new lures added) between the 18th and 20th of May. The traps were emptied a second time between the 7th and 11th of June. Similarly, the traps with 3-hydroxy-2-hexanone were deployed on the 28th of April, emptied a first time on the 20th of May, with new lures added simultaneously. The traps were emptied a second time on the 11th of June and the trapping discontinued.

### Statistical Analysis

Data from the bioassay did not follow a normal distribution and had nonhomogeneous variances between the treatment groups. Hence, we used the nonparametric *Kruskal-Wallis H test* (using mean ranks) for statistical comparisons between groups of three or more treatments and the *Mann-Whitney U test* for pairwise post hoc comparisons. We applied a Holm-Bonferroni correction (Holm 1979) to control for the risk of making a type one error when performing multiple tests with the *Mann-Whitney U test*. The *Mann-Whitney U test* was also used to compare the average number of females and males per trap with different pheromone blends. Statistical significance was defined as probability values (*P*), and adjusted probabilities (post hoc tests), lower than 0.05. All calculations were performed in IBM® SPSS® Statistics, version 24.0 for Windows, 64-bit edition (IBM Corp. 2016).

## Results

### Pheromone Identification

**Fig. 1:**
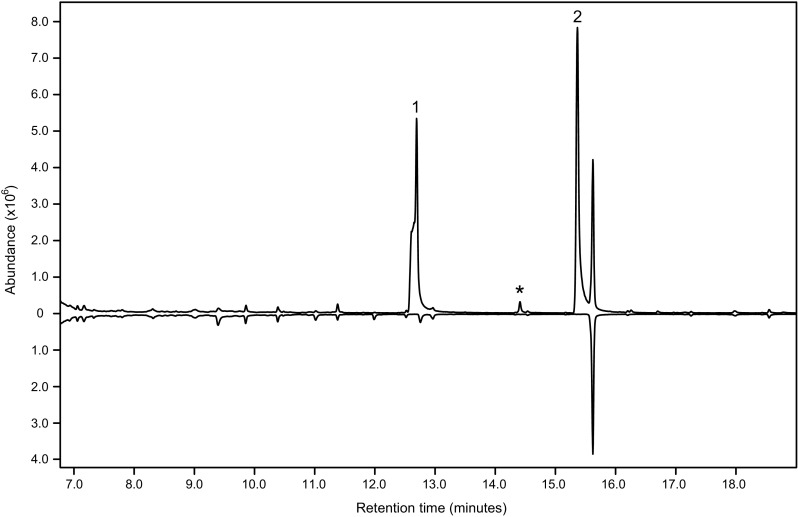
Total ion chromatograms of aeration samples (DB-WAX column) of headspace volatiles produced by adult male (top trace) and female (bottom, inverted trace) *Phymatodes pusillus* ssp. *pusillus*. Male-specific compounds are 2-methyl-1-butanol (1), 1-hexanol (2) and 2-methyl-1-pentanol (*). The system contaminant present in both samples at 15.7 min is diacetone alcohol. The shoulder on the front of peak 1 is a chromatographic artefact also observed with synthetic 2-methyl-1-butanol

**Fig. 2:**
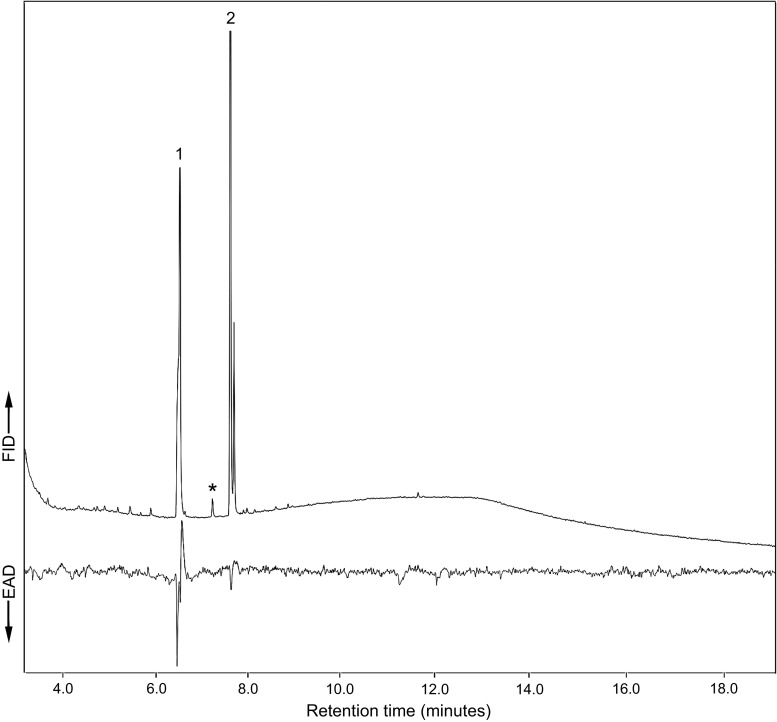
Representative GC-EAD analysis of volatiles from male *Phymatodes pusillus* ssp. *pusillus*. Top trace shows the GC chromatogram and the bottom trace displays the EAD response of an antenna of a conspecific female. Responses to 2-methyl-1-butanol (1) and 1-hexanol (2) were recorded, but not to 2-methyl-1-pentanol (*)

**Fig. 3:**
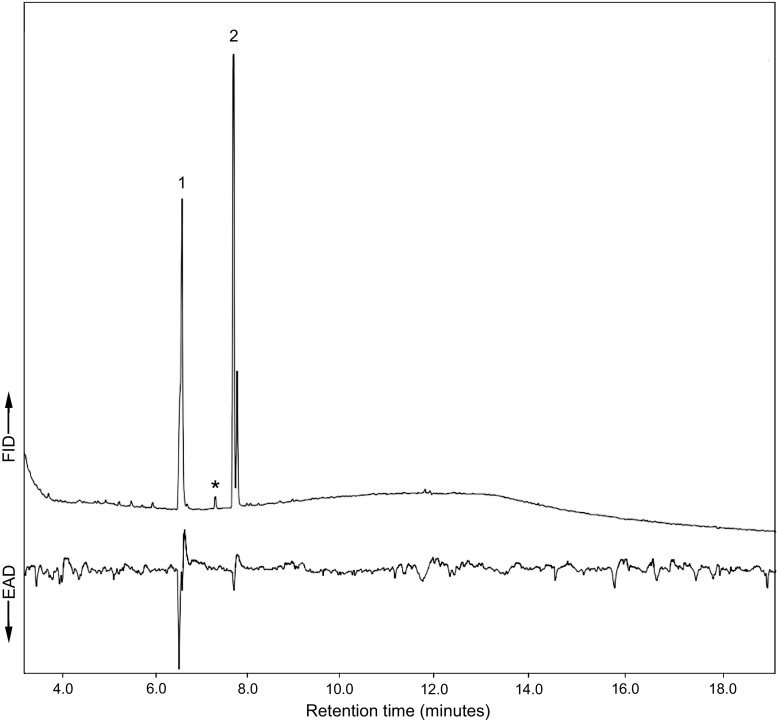
Representative GC-EAD analysis of volatiles from male *Phymatodes pusillus* ssp. *pusillus*. Top trace shows the GC chromatogram and the bottom trace displays the EAD response of an antenna of a conspecific male. Responses to 2-methyl-1-butanol (1) and 1-hexanol (2) were recorded, but not to 2-methyl-1-pentanol (*). Other apparent responses were not consistently observed in different recordings

Analysis of the aeration samples on the two GC-MS setups revealed that two compounds were consistently present in large quantities in the extracts of volatiles from male beetles, but completely absent in all extracts from females and blank controls (Fig. 1). Matching the two male-specific compounds to the database libraries indicated that the compounds were 1-hexanol and 2-methyl-1-butanol, and this was confirmed by comparing their retention times and mass spectra with those of synthetic standards. The relative proportions of 1-hexanol and 2-methyl-1-butanol fluctuated. 2-Methyl-1-butanol was clearly the minor component in four samples (10–18% of the quantity of 1-hexanol), but in two samples it reached 96 and 109% relative to 1-hexanol. Female and male antennae reproducibly responded to both 1-hexanol and 2-methyl-1-butanol in GC-EAD analyses (Figs. 2, 3), usually with a stronger response to the 2-methyl-1-butanol compared with that to the 1-hexanol. No reproducible response to any other compound in the extracts from male beetles was observed.

Analyses of samples from female beetles did not show any female-specific compound that was present in more than one sample when compared to analyses of samples from male beetles and the blank control. Further, volatiles from male and female beetles did not show any common compound which was absent in the volatiles from the corresponding blank controls. However, in three of the six extracts of volatiles from males, a third male-specific compound was identified as 2-methyl-1-pentanol, verified by comparing with a synthetic standard. The compound was present in small quantities of 1–3% of the quantity of 1-hexanol and no response to it was observed in three GC-EAD analyses of the corresponding samples.

### Field Bioassays

**Fig. 4:**
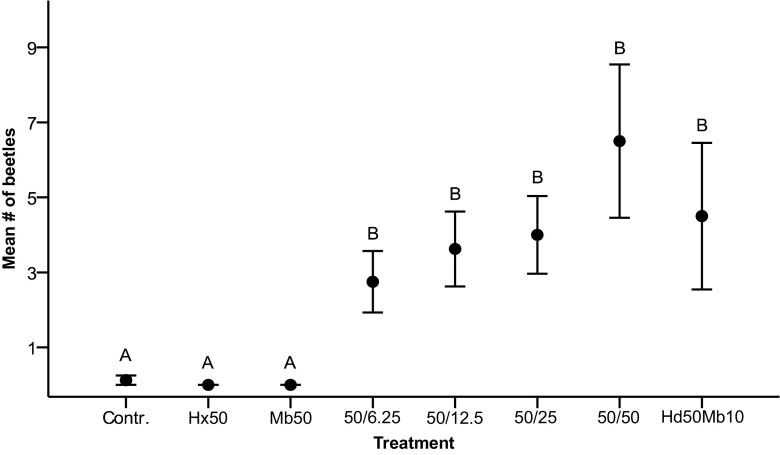
Mean (± 1 SE) number of male and female *Phymatodes pusillus* ssp. *pusillus* captured per collection date in traps baited with different lure compositions (*N* = 8 samples); blank control (isopropanol), 1-hexanol (50 mg), 2-methyl-1-butanol (50 mg), four blends with 1-hexanol (50 mg) and an increasing amount of 2-methyl-butanol (6,25–50 mg) and a blend of 3-hydroxy-2-hexanone (50 mg) and 2-methyl-1-butanol (10 mg). Means with different letters are significantly different (*P* < 0.05)

A total of 172 individuals of *Phymatodes p. pusillus* were captured in the field bioassay. Considering both sexes combined, *P. p. pusillus* showed a statistically significant attraction to all four blends of 1-hexanol and 2-methyl-1-butanol compared to the blank control (Fig. 4). 1-Hexanol and 2-methyl-1-butanol were not attractive as single components to the beetles and catches did not differ from the control (*Kruskal-Wallis*: *H* = 2.0, 2 d.f., *P* = 0.368). There was a trend that a higher proportion of 2-methyl-1-butanol in the blend increased attraction, but the differences were not statistically significant when comparing the four blends (*Kruskal-Wallis*: *H* = 2.04, 3 d.f., *P* = 0.565). The blend of 3-hydroxy-2-hexanone and 2-methyl-1-butanol also elicited significant attraction compared to the blank control (Fig. 4), but the catch was not significantly different from those with any of the four blends of 1-hexanol and 2-methyl-1-butanol (*Kruskal-Wallis*: *H* = 1.83, 4 d.f., *P* = 0.768).

As catches were female-biased in the 2016 bioassay, we also analyzed attraction to the different treatments with females and males considered separately. Compared to the control, males were significantly attracted to the two blends with a higher quantity of 2-methyl-1-butanol (25 mg/bait *U test*: *U* = 8.0, *P* = 0.003 and 50 mg/bait *U test*: *U* = 12.0, *P* = 0.01), as well as to the heterospecific blend of 3-hydroxy-2-hexanone and 2-methyl-1-butanol (*U test*: *U* = 14.5, *P* = 0.034). The two blends with a lower proportion of 2-methyl-1-butanol captured a higher number of male beetles than the control, but the differences were not significant (6.25 mg/bait *U test*: *U* = 20.0, *P* = 0.064 and 12.5 mg/bait *U test*: *U* = 24.0, *P* = 0.144). Females were significantly attracted to all four blends of 1-hexanol and 2-methyl-1-butanol compared to the control (6.25 mg/bait *U*-test *U* = 10.0, *P* = 0.011; 12.5 mg/bait *U* = 5.5, *P* = 0.003; 25 mg/bait *U* = 9.5, *P* = 0.009 and 50 mg/bait *U* = 9.0, *P* = 0.008), as well as to the heterospecific blend (*U test*: *U* = 14.5, *P* = 0.034).

**Fig. 5:**
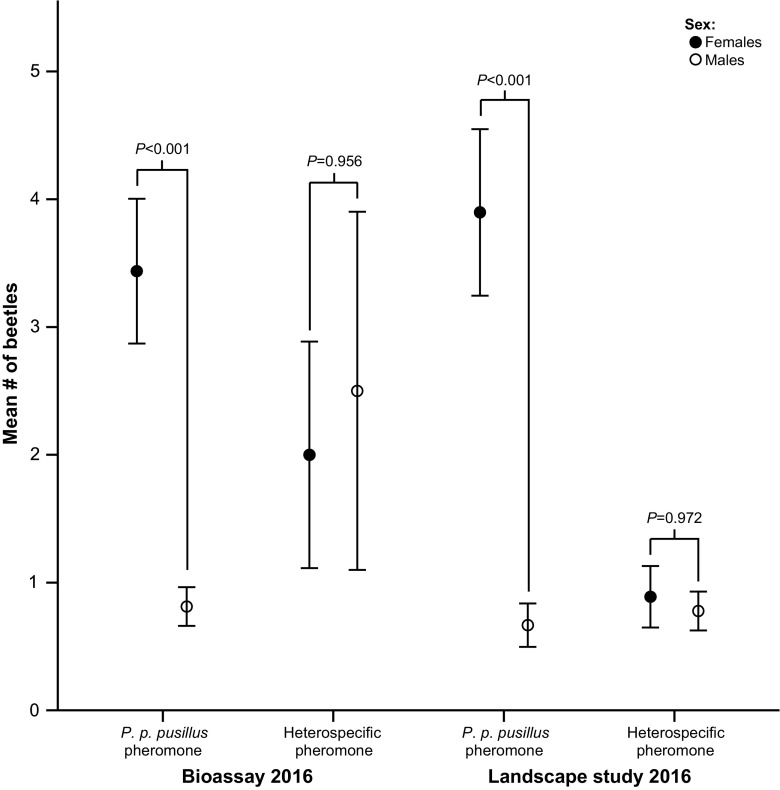
Mean number (± 1 SE) of female and male *Phymatodes pusillus* ssp. *pusillus* captured per trap and collection date during the bioassay and a larger landscape study. For the bioassay, all beetles captured on the four different blends of 1-hexanol and 2-methyl-1-butanol have been pooled. The pheromones used in the landscape study are a blend of 1-hexanol (50 mg) and 2-methyl-1-butanol (25 mg) and the heterospecific pheromone a blend of 3-hydroxy-2-hexanone (50 mg) and 2-methyl-1-butanol (10 mg). Mean number of female and male beetles were compared with the *Mann Whitney U test*

The traps with the four different blends of the *P. p. pusillus* pheromone attracted a significantly higher average number of female beetles per trap compared to the average number of male beetles per trap (Fig. 5). The percentage of females captured on the four blends combined was 81% (110 females and 26 males), with females representing 75% (total 32 beetles, blend of 25 mg 2-methyl-1-butanol), 82% (22 beetles, 6.25 mg), 83% (52 beetles, 50 mg) and 86% (29 beetles, 12.5 mg) of the total catch. In contrast, the numbers of females and males captured per trap were not significantly different with the heterospecific blend of 3-hydroxy-2-hexanone and 2-methyl-1-butanol (16 females and 19 males in total) (Fig. 5). A similar pattern was observed in the data from the 2016 landscape study. The average number of female beetles captured per trap was significantly higher than that of the males among the traps with lures containing the *P. p. pusillus* pheromone (Fig. 5) and females represented 86% of the total number of individuals (152 females and 26 males in total). Again, there was no significant difference in the average number of females and males captured per trap in traps baited with the heterospecific pheromone blend (16 females and 14 males in total).

The traps which tested 3-hydroxy-2-hexanone as a single component in 2017 captured a single individual of *P. p. pusillus* (trap average: 0.08 beetles). In comparison, the heterospecific blend of 3-hydroxy-2-hexanone and 2-methyl-1-butanol was significantly more attractive and captured a total of 37 individuals (trap average: 1.03 beetles) (*Mann Whitney U test*: *U* = 169.0, *P* < 0.001). The sex ratio for the heterospecific blend was 51% females and 49% males.

## Discussion

The significant attraction of both male and female *P. p. pusillus* to blends of the male-produced compounds 1-hexanol and 2-methyl-1-butanol in the field bioassay, coupled with reproducible antennal responses to the two compounds in GC-EAD, demonstrates that *P. p. pusillus* utilizes a blend of 1-hexanol and 2-methyl-1-butanol as a male-produced, long-distance aggregation-sex pheromone. In contrast, when the compounds were tested separately, no attraction was observed. Unlike Schröder (1996), we found no trace of 1-butanol in the extracts of volatiles from male beetles as verified with a synthetic standard. Schröder (1996) also reported traces of 1-octanol as a compound specific to extracts from males, but we only found a trace of 1-octanol (0.4% of the amount of 1-hexanol) in one of our extracts from male beetles and no response to it was documented in the GC-EAD studies (two of the successful recordings used the extract of volatiles from male beetles that contained 1-octanol). We have not been able to explore the causes of these discrepancies. One possible reason could be that different populations of the species use different pheromone compounds and/or proportions, which has been observed in some species of moths and bark beetles (Lanier et al. 1980; Löfstedt et al. 1986). However, this appears unlikely as both studies examined the same subspecies and populations that are less than 600 km apart. The specific subspecies is not indicated in the work by Schröder (1996), but the beetles originated from the Hamburg-region (F. Schröder pers. comm. 2018) and only the nominal subspecies, *P. pusillus* ssp. *pusillus*, is known to occur in Germany (Klausnitzer et al. 2016).

The relative amounts of 1-hexanol and 2-methyl-1-butanol produced by the male beetles varied from 100:10 to 100:109 respectively. Consistent with this, trap catches with the four blends of 1-hexanol and 2-methyl-1-butanol were not significantly different, probably at least in part due to different numbers of beetles being present at the different sampling locations. There was a trend to higher catches with a higher proportion of 2-methyl-1-butanol, but further studies are needed to verify this.

*Phymatodes p. pusillus* belongs to the Cerambycinae subfamily and so far only male-produced pheromones are known from previous studies of species in this subfamily (Hanks and Millar 2016; Millar and Hanks 2017). A total of about 30 species in the subfamily have confirmed behaviorally active, male-produced aggregation-sex pheromones (Millar and Hanks 2017). Typically, the pheromones consist of one or two compounds and the compounds identified here as the pheromone of *P. p. pusillus* fit well with the general structural motifs that are known to occur within the subfamily. 2-methyl-1-butanol has been reported as a pheromone, or pheromone component, of several closely related species within the same tribe (Callidiini), but no other cerambycid species are known to use 1-hexanol (see Millar and Hanks 2017). This compound may be relatively species specific to *P. p. pusillus*, although further studies on other species are needed*.* As observed in this study, when species produce blends of compounds their attraction is often significantly enhanced when the two pheromone components are applied as a blend, compared to application of the components as single compounds (e.g. Millar and Hanks 2017; Mitchell et al. 2015; authors unpubl. data).

We could not detect 3-hydroxy-2-hexanone in any extract of volatiles from either male or female *P. p. pusillus* beetles. As demonstrated in our bioassay in 2016 and the additional trapping in 2017, 2-methyl-1-butanol and 3-hydroxy-2-hexanone are not attractive to *P*. *p. pusillus* as single components and the attraction to the blend of 2-methyl-1-butanol and 3-hydroxy-2-hexanone is a truly synergistic effect of the latter compound.

3-Hydroxy-2-hexanone and 2-methyl-1-butanol are two of the most common constituents of pheromones of other species in the Cerambycinae subfamily (see overview in Millar and Hanks 2017). It is not unusual to trap other species of cerambycids in traps baited with a specific pheromone intended to catch one particular species. This is due to the fact that many species have pheromones consisting of two or three compounds where one compound can be shared by multiple species, and the beetles frequently show some limited attraction to single components even if not all components of a species’ full blend are present in the lures. This type of cross-attraction has been observed in several studies (e.g. Hanks et al. 2007; Lacey et al. 2009). However, the attraction is usually weak when one or more components is missing and no previous studies that we are aware of have demonstrated the phenomenon that a heterospecific component can significantly synergize attraction to a component of the species’ own pheromone that is inactive when applied as a single compound. Previous studies have only demonstrated that a significant synergy in attraction is obtained when combining conspecific components.

It has been proposed that male-produced aggregation-sex pheromones are often utilized in conjunction with sparse and aggregated larval substrates that constitute a potential resource for females (Landolt and Philips 1997; Schlyter and Birgersson 1999). In this context, heterospecific cross-attraction could be beneficial for locating precisely the types of transient substrates that occur sparsely and are only suitable to the larvae for a short period of time (one or two years), on which many cerambycid species depend. Regarding *P. p. pusillus* and its attraction to the blend of 3-hydroxy-2-hexanone and 2-methyl-1-butanol, this specific combination is used by at least two other closely related and sympatric species; *Pyrrhidium sanguineum* and *Poecilium alni* (Winde et al. manuscript in prep.). In fact, *P. p. pusillus* and the two species mentioned above have nearly identical overlap in adult phenology (Lindhe et al. 2010), utilize oak wood of similar and partly overlapping dimensions and can often be observed together (authors pers. observations). The pheromone of *P. sanguineum* and *P. alni* could thus function as a host cue for *P. p. pusillus*, signaling the location of suitable substrates for oviposition. A similar observation was made by Hanks et al. (2007) who found that males of the North American Cerambycinae species *Phymatodes grandis* (*P. lecontei* in the publication by Hanks et al.) only produced 2-methyl-1-butanol and was significantly attracted to traps with this compound, but also to traps baited with 3-hydroxy-2-hexanone as a single component. Hanks et al. (2007) hypothesized that 3-hydroxy-2-hexanone could function as host cue for *P. grandis* as another species, *Xylotrechus nauticus*, is known to produce this compound and share the same host trees as *P. grandis*.

We also observed a marked difference in the relative numbers of males and females of *P. p. pusillus* that are captured in traps with the species’ own pheromone and in traps with lures of the heterospecific blend of 3-hydroxy-2-hexanone and 2-methyl-1-butanol (Fig. 5). The species’ own pheromone captures a significantly higher relative number of females, while essentially equal numbers of females and males are captured with the heterospecific pheromone blend. The biological significance of this sex-associated difference can only be speculated on here, but it has been argued that the function of male aggregation-sex pheromones among cerambycids is primarily to bring the two opposite sexes together for the purpose of mating, not to initiate aggregation behavior, which makes aggregation-sex pheromones in longhorn beetles more similar to traditional sex pheromones than an aggregation pheromone (see Cardé 2014; Millar and Hanks 2017). This could explain why females are more attracted to the species’ own pheromone than males. However, under certain conditions it may be beneficial also for males to respond to the species’ own aggregation-sex pheromone, for instance when a male has been unable to attract a female on his own, or locate suitable substrates where females can be encountered more easily. Such a situation could arise comparatively often for species that are dependent on short-lived substrates that fluctuate significantly in space and time (fresh, dead branches). Under such conditions, response to the aggregation-sex pheromone by males may be used as a secondary approach to increase the probability for males to come into contact with females and thereby increase male fitness. In contrast, the blend of 3-hydroxy-2-hexanone and 2-methyl-1-butanol, that the sympatric species use, may function as a general host cue for both male and female *P. p. pusillus*, indicating locations where females can find oviposition sites and where males are more likely to encounter females. This could explain the shift towards the equal numbers of males and females that are captured with the heterospecific blend of 3-hydroxy-2-hexanone and 2-methyl-1-butanol (Fig. 5).

The ecology and conservation requirements of *Phymatodes p. pusillus* have been practically impossible to study quantitatively under natural conditions in the field due to the species’ dependency on a transient substrate, which occurs sporadically and often out of reach high up in the tree tops. Merely to detect the species at sites where it is present is a difficult and time-consuming undertaking. Conversely, it is equally difficult to prove that an area is not inhabited by the species as it can easily remain undetected. Consequently, there is no information available on how populations of *P. p. pusillus* are developing in response to the extensive environmental changes that are taking place, such as climate change and the rapidly increasing harvest of forest resources for biofuel production. Being primarily dependent on oak branches of thin diameter, the species has until recently been able to utilize thinning and cutting residues left by forestry following management actions in oak forests. However, the expansion of the practice to collect almost all the biomass including thin branches and twigs for biofuel production may affect the species negatively as large amounts of suitable larval substrate are removed from the forest. Additionally, stacks of biofuel material (typically fresh branches and twigs), such as the one where we gathered our wood substrates, are likely to act as so-called ecological traps for the species (see e.g. Hedin et al. 2008). The pheromone that was identified in the present study should be eminently suitable to survey presence and absence of the species at individual sites and to monitor populations over time. The effectiveness of the pheromone is indicated by the fact that more than five times as many individuals of *P. p. pusillus* were captured in the bioassay 2016, as have been hand-collected in Sweden over the last 60 years (total of about 35 individuals). Furthermore, besides detection and monitoring, pheromone-based trapping could also be used as a general tool to conduct future large scale quantitative autecological and conservation orientated studies associated with the abovementioned environmental issues. Development of a quantitative method to survey and monitor the species was recommended as part of the national action plan for its preservation in Sweden (Franc 2013), but the pheromone should prove useful also in an international context, as the species is rare and local over much of continental Europe and the pheromone components 1-hexanol and 2-methyl-1-butanol are inexpensive and commercially available.
